# Reuse of healing abutments: Ethical, biological and professional training implications

**DOI:** 10.4317/jced.59831

**Published:** 2022-10-01

**Authors:** Gustavo Paganotto, Roberto Zimmer, Celso-Afonso Klein-Junior, Elken-Gomes Rivaldo

**Affiliations:** 1 Postgraduate Program in Dentistry, Lutheran University of Brazil (ULBRA), Canoas, Brazil

## Abstract

**Background:**

The aim was to estimate the prevalence of reuse of healing abutments, the methods used in disinfection and to analyze the reasons that lead to the reuse of these components by professionals who work in rehabilitation with dental implants.

**Material and Methods:**

For this, an online data collection was carried out through a questionnaire developed in Google Forms. This questionnaire was applied to 284 specialists in implantology, randomized, of the 1,147 registered in the Regional Council of Dentistry of Rio Grande do Sul. The questionnaire was divided into three parts: the first containing the Free and Informed Consent Form; the second referring to the correspondents’ demographic data; and the third part with information on reuse, disinfection and sterilization routines used, risk perception and information from manufacturers. To estimate the prevalence in the reuse of healing abutments by implantologists, the frequency of responses was used.

**Results:**

The results showed that almost all implantologists reuse healing abutments (98.1%). The main reasons for reuse were cost (71.2%) and practicality (26%). Regarding the limitations, 53.3% do not see limitations in its reuse, 20% associate it with increased roughness, 17.8% with the accumulation of organic matter and 8.9% with cross-infection as limitations for reuse. Already 95.3% did not receive any guidance from manufacturers on the reuse of these components. Enzymatic detergent and ultrasonic bath was the most used cleaning method (50.7%) followed by ultrasonic bath (23.3%). Autoclave was the method used for sterilization for all respondents.

**Conclusions:**

The reuse of healing abutments is a practice adopted by implantologists in the state of Rio Grande do Sul, Brazil and most professionals do not observe limitations in this practice since these components are used repeatedly. Decontamination with enzymatic detergent and an ultrasonic bath is the most commonly used procedure associated with autoclave sterilization.

** Key words:**Abutment, reuse, decontamination, sterilization.

## Introduction

In rehabilitation with dental implants, healing abutment components play an important role in conducting the repair and stabilization of peri-implant tissues. Once installed, healing abutments provide adhesion of epithelial tissue cells, forming a physical barrier against bacterial colonization at the implant/healing abutment component junction (1). Although it is a temporary use component, its permanence time in the mouth can vary from a few weeks to several months, maintaining close contact with oral fluids, microbiota and organic matter (2). Therefore, healing abutments are subject to the formation of a strongly adhered biofilm where several bacterial and fungal species can reside (3).

In order for the healing abutments to properly exercise their purpose, a decontamination process of this component must be carried out, which consists of the complete removal of organic matter and sterilization, completely eliminating all forms of viral, bacterial and fungal activity (2,4,5). Several decontamination methods are used, however, studies point to the presence of residual organic matter and even the presence of viable bacterial activity (3,4,6,7). It is suggested that the permanence of organic matter and also the maintenance of bacterial viability would entail biological risks, such as contamination of the implant/healing abutment junction and peri-implant tissues which would imply in the impossibility of reusing these components (2,4,8).

In addition to the remaining contamination, reuse causes changes on the surfaces of healing abutments, increasing the porosity of the coating layer and facilitating bacterial colonization at the junction between the healing abutment and the implant. This fact can induce a chronic inflammatory process in the peri-implant tissues (2). Even with the possibility of risk to the success of the treatment, studies report that professionals routinely reuse healing abutments and the main justification for this practice would be the economic factor (5,9-11).

Therefore, due to the great possibility of failures in the cleaning and sterilization process, risk of bacterial contamination and structural changes of healing abutments, knowledge about the routine of professionals in handling these components is necessary. Likewise, it is extremely important to understand their perception of the limitations and risks inherent in the reuse of these healing abutments. Thus, the objective of the present study was to estimate the prevalence of reuse of healing abutments, the most used methods for decontamination and sterilization of these devices and to analyze the reasons that lead implantologists in Rio Grande do Sul to reuse these materials.

## Material and Methods

The present study was approved by the Ethics and Research Committee of the Universidade Luterana do Brasil (opinion number: 4,943,279) and participants were included in the study after signing the Free and Informed Consent Form.

The sample size calculation was performed in the GPower 3.1.9.4® Program based on a population of 1,147 implantologists with active status in the Regional Council of Dentistry of the state of Rio Grande do Sul, Brazil (CRO/RS). Initially, the need for 203 participants was estimated, evenly distributed, with a sampling error of 5.0% and a confidence interval of 95%. Added to this number was a forecast of non-respondents of 40%, totaling 284 selected professionals.

The professionals were randomized using the Research Randomizer software. Dentists who were inactive or with low CRO/RS for any reason were excluded from the study, and the name drawn was replaced by the next name on the list.

 Data collection began in September 2021 and was carried out through an online questionnaire sent by email and WhatsApp. The professional who did not show any return after 7 days received the questionnaire again, totaling a maximum of two submissions. The questionnaire was made available via Google Forms and conFigured not to store the participants’ personal data, only the answers.

The questionnaire was divided into three parts: the first containing the Free and Informed Consent Form; the second referring to the correspondents’ demographic data (9 questions); and the third part with information on reuse, time of use in the maxilla and mandible, disinfection and sterilization routines used, risk perception and information from manufacturers (13 questions). The collection of responses ended in November 2021.

To assess the internal consistency of the questionnaire, Cronbach’s Alpha was used, where a value between 0.7 and 0.8 was obtained, which was considered acceptable (12). To estimate the prevalence in the reuse of healing abutments by implantologists, the frequency of responses was used. The Shapiro-Wilk normality test was also performed, verifying that the collected data are normal and, later, the Pearson’s coefficient was performed to verify if there would be a correlation with the non-reuse of healing abutments and the perception of risk in the reuse of these components. All statistical tests were performed in the SPSS program version 3.1.9.4®.

## Results

A total of 202 responses were obtained, consisting of 75.2% men and 24.8% women. The mean age was 42.7 years with a standard deviation of 8.4 years. Most respondents were between 41 and 50 years old (45.5%). Regarding the origin of the undergraduate degree in dentistry, most respondents come from private institutions (57.4%), while 42.6% came from public institutions. With regard to degrees, 53% of implantologists have more than one specialization, while 31.2% have a specialization only in implantology, 10.4% have a master’s degree and 5.4% have a doctorate.

Implantology professionals work mostly (74%) both in the surgical area and in the area of prosthesis on implants. Regarding the place of work, most work in their own private practice (89.6%), followed by third-party consultation (9.2%) and 1.2% in the military service.

**Figure 1:**
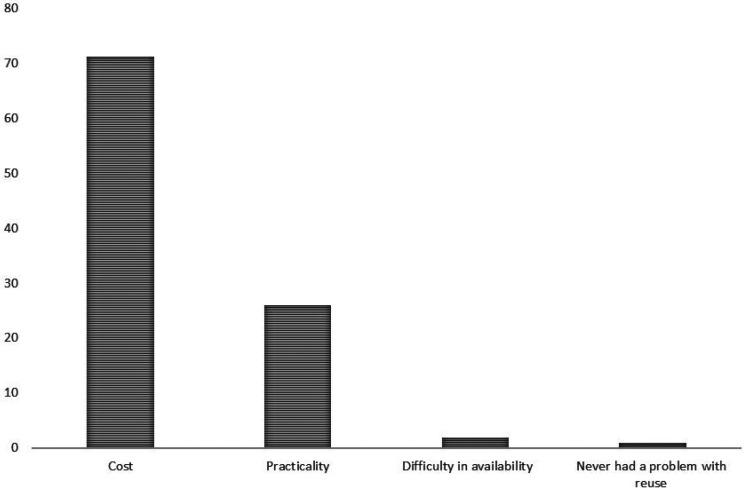
Main reasons to reuse healing abutment components.

The reuse of healing abutments is a practice adopted by 98.1% of respondents, while only 1.9% do not reuse. When asked the main reason for reusing, 71.2% answered that it was cost, while 26% related to practicality and 1.9% to difficulty in availability (Fig. 1).

Reusing countless times is a routine adopted by 98.1%, while only 8.2% reuse only once. When asked about communicating to the patient about the reuse of components, 94.5% of implantologists do not communicate, and only 5.5% reported informing. All respondents stated that they disinfect the healing abutments after use.

**Figure 2:**
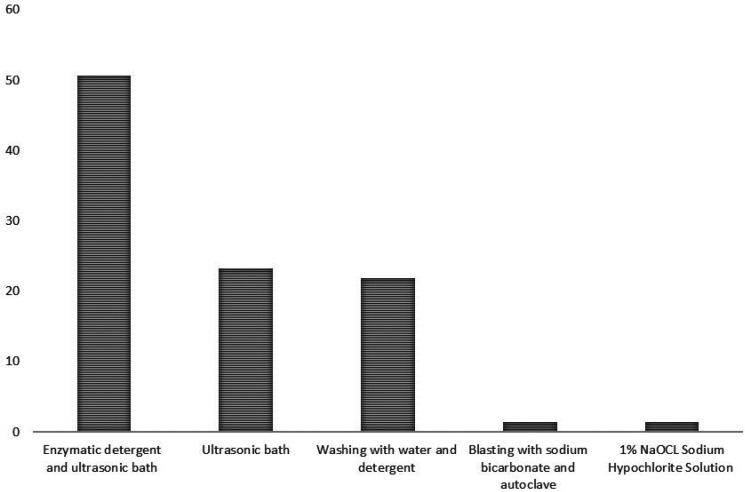
Procedures used in cleaning/disinfection.

Most of the procedures used in the disinfection of the components consist of enzymatic detergent and an ultrasonic bath (50.7%), 23.3% use only an ultrasonic bath and 21.9% washing with water and detergent, 1.4% blasting with sodium bicarbonate and 1.4% sodium hypochlorite solution (Fig. 2).

For sterilization, all professionals use an autoclave and none declared using 2% glutaraldehyde. They were also asked about receiving guidance from manufacturers on the reuse of healing abutments, however, 95.3% of respondents claimed not to have received this type of information.

**Figure 3:**
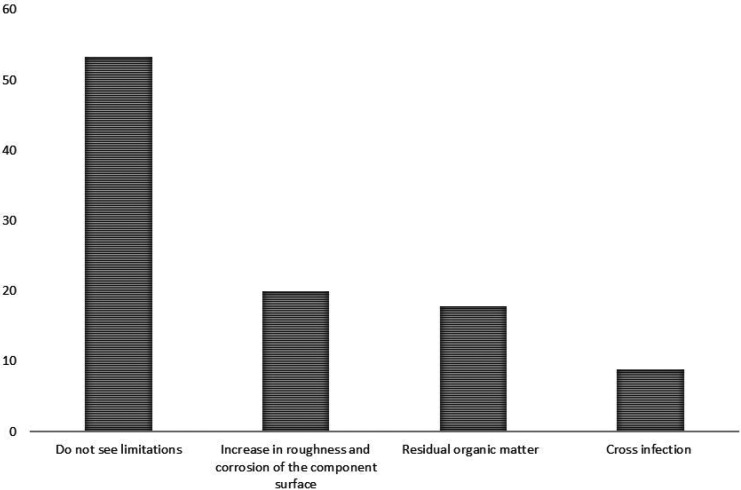
Limitations associated with the reuse of healing abutments.

When asked about the limitations associated with the reuse of healing abutments, 53.3% of the respondents did not see limitations, while 20% associated it with increased roughness and corrosion of the surface of the component, 17.8% with the presence of remaining organic matter and 8.9% with cross infection (Fig. 3). There was no correlation between the guidelines provided by the manufacturers with possible implications for the reuse of healing abutments.

## Discussion

The reuse of healing abutments is a practice adopted by 98.1% of implantologists registered at CRO/RS. The results found corroborate the information reported by several authors (5,11,13,14). The cost factor is the main reason that leads professionals to reuse healing abutment components. However, this reason is extremely controversial with regard to safety and ethics for the patient (11), which perhaps explains why 95.1% of professionals do not inform the patient that they reused the component.

For the reuse of these components, several cleaning procedures were reported, with 50.07% using enzymatic detergent and ultrasonic cuvettes as a procedure for removing organic matter. However, the use of enzymatic detergent and ultrasonic cuvettes has shown limited results in the complete removal of organic matter (4,14,15). In another portion, 23.3% of professionals reported using only the ultrasonic cuvette for cleaning and 21.9% use only washing with water and detergent, but the effectiveness of these two methods is extremely low (9.16), with contamination of organic matter residual in 95% of the samples (8). All professionals use autoclave as a sterilization method. However, with the permanence time of these components in contact with organic matter and oral fluids ranging from 30 to 180 days and, still, with the reuse being performed repeatedly (91.8%), a compact biofilm colonized by a viable microbiota can form and strongly adhere to the component (10), and therefore, cannot be completely removed by the methods described (16-18).

The healing abutments subjected to repeated chemical, mechanical and physical processes to provide their reuse may undergo surface changes such as oxidation of the titanium coating layer, thus creating porosities (6,19). These porosities are an additional component for the formation of a complex biofilm that provides the adhesion of several bacterial species, including those present in the pathological processes of peri-implant tissues (3). Other factors such as cleaning performed by the patient, multiple implantations and anatomical characteristics may contribute to biofilm retention.

This presence of organic matter and contamination after reusing the healing abutments represents an alarming biological risk (20,21), which is perceived by only 17.8% of the interviewees, which may explain why 53.3% of the professionals reported that there was no risks for reuse. This result may be strongly related to the lack of information on the correct use of these components, since 95.3% of respondents claimed not to have received any guidance from the manufacturers. Additionally, most of the respondents have a consolidated training in the specialty, 53% of the respondents have more than one specialty, however, professionals have a low perception of the biological risk in the reuse of healing abutments, which shows the lack of information in the training of the specialist in implantology.

Several studies show that there is a high possibility of contamination of healing abutments even after cleaning procedures (4,8-10,14,16,19). As elucidated in the present study, professionals involved in treatment with dental implants reuse healing abutments and have minimal perception of the risks. It is therefore important that the results of the present study can contribute to the understanding of the limitations and biological risks present in the reuse of these components, in addition to promoting the discussion about the effectiveness of the procedures used in the decontamination of these critical articles.

It can be concluded that implantologists in the state of Rio Grande do Sul, Brazil reuse healing abutments, and for this purpose, they use a variety of cleaning procedures and autoclave sterilization. The lack of perception about the risks and any guidance from the manufacturers predominate in the training and conduct of the specialists participating in the present study.

## References

[1] Canullo L, Menino M, Santori G, Rakic M, Sculean A, Pesce P (2020). Titanium abutment surface modifications and peri-implant tissue behavior: a systematic review and meta-analysis. Clin Oral Investig.

[2] Jain SS, Sareda TJS, Danyal AS, Wenwen H, Kelli LP, Thomas G (2020). Effects of multiple implantations of titanium healing abutments: Surface characteristics and microbial colonization. Dent Mater.

[3] Wheelis SE, Wison Jr GT, Valderrama P, Rodrigues DC (2018). Surface characterization of titanium implant healing abutment before and after placement. Clin Implant Dent Relat Res.

[4] Barreiros P, Braga J, Faria-Almeida R, Coelho C, Teugher W, Souza JCM (2020). Remnant oral biofilm and microorganisms after autoclaving sterilization of retrieved healing abutments. J Periodotics Res.

[5] Cakan U, Delibasi CE, Kivanic M (2015). Is it safe to reuse dental implant healing abutments sterilized and serviced by dealers of dental implant manufactures? An in vitro sterility analysis. Implant Dent.

[6] Jain SS, Siddiqui DA, Wheelis SE, Palmer KL, Thomas GW, Rodrigues DC (2021). Mammalian cell response and bacterial adhesion on titanium healing abutments:effect of multiple implantation and sterlization cycles. Clin Oral Investig.

[7] Zhao B, van der Mei HC, Rustema-Abbing M, Busscher HJ, Ren Y (2015). Osteoblast integration of dental implant materials after challenge by sub-gingival pathogens: a co-culture study in vitro. Int J Oral Sci.

[8] Stachi C, Berton F, Porrelli D, Lombardi T (2018). Reuse of Implant Healing abutments: comparative evaluation of efficacy of two cleaning procedures. Int J Prosthodont.

[9] Wadhwani C, Schonnenbaum TR, Audia F, Chung KH (2016). In vitro study of the contamination remaining on used healing abutments after cleaning and sterilizing in dental practice. Clin Implant Dent Relat Res.

[10] Almehmadi AH (2021). An in vitro analysis of Sodium Hyplochlorite decontamination for the reuse of implant healing abutments. J Oral Implantol.

[11] Avinash BS, Swati K, Kruttika B (2020). Should Healing Abutments and Cover Screws for Dental Implants be reused? A systematic Review. J Prosthodont.

[12] Bujang MA, Omar ED, Baharum NA (2018). A review on sample size determination for Cronbach's alpha test: a simple guide for researchers. Malays J Med Sci.

[13] Chew M, Tompinks G, Tawise-Smith A, Waddel JMS (2018). Reusing titanium healing abutments: comparison of two decontamination methods. Int J Prosthodont.

[14] Sanchez-Garcés MA, Jorba M, Ciurana J, Vinas M, Vinuesa MT (2019). Is the re-use of sterilized implant abutments safe enough? (Implant abutment safety). Med Oral Patol Oral Cir Bucal.

[15] Narvekar A, Estepa AV, Naqvi A, Nares S (2020). Used dental implant healing abutments elicit immune responses: A comparative analysis of detoxification strategies. Clin Implant Dent Relat Res.

[16] Eswaramurthy P, Aras M, DSouza KM, Nagarsekar A, Gaunkar RB (2021). Contemporay sterilization protocols of healing abutments for reusability: A systematic review. JDR Clin Trans Res.

[17] Ferronato D, Fumagalli D, Farah A, Rasperini G (2018). Decontamination of Customized Laser-Microtextured Titanium abutment: A comparative in vitro study of different cleaning procedures. Int J Periodontics Restorative Dent.

[18] Canullo L, Massuci L, Quaranta G, Patini R, Caponio VCA, Pesce P (2021). Culturomic and quantitative real-time-polymerase chain reaction analyses for early contamination of abutments with different surfaces: A randomized clinical trial. Clin Implant Dent Relat Res.

[19] Kyaw TT, Hanawa T, Kasugai S (2020). Investigation of different electrochemical cleaning methods on contaminated healing abutments in vitro: an approach for metal surface decontamination. Int J Implant Dent.

[20] Vezeau PJ, Keller JC, Wightman JP (2000). Reuse of Healing Abutments: an in vitro model of plasma cleaning and common sterilization techniques. Implant Dent.

[21] Charalampakis G, Ramberg P, Dahlen G, Berglundh T, Abrahamsson I (2015). Effect of cleasinf biofilm formed on titanium discs. Clinical Oral Implants Res.

